# Differences in Grain Yield and Nitrogen Uptake between Tetraploid and Diploid Rice: The Physiological Mechanisms under Field Conditions

**DOI:** 10.3390/plants13202884

**Published:** 2024-10-15

**Authors:** Jian Xiao, Zhuang Xiong, Jiada Huang, Zuolin Zhang, Detian Cai, Dongliang Xiong, Kehui Cui, Shaobing Peng, Jianliang Huang

**Affiliations:** 1 National Key Laboratory of Crop Genetic Improvement, Ministry of Agriculture Key Laboratory of Crop Eco-Physiology and Farming System in the Middle Reaches of the Yangtze River, College of Plant Science and Technology, Huazhong Agricultural University, Wuhan 430070, China; xiaoj202410@163.com (J.X.); dlxiong@mail.hzau.edu.cn (D.X.); cuikehui@mail.hzau.edu.cn (K.C.); speng@mail.hzau.edu.cn (S.P.); 2 Guangxi Key Laboratory of Plant Functional Phytochemicals and Sustainable Utilization, Guangxi Institute of Botany, Guangxi Zhuang Autonomous Region and Chinese Academy of Sciences, Guilin 541006, China; xiongzhuang@gxib.cn; 3 College of Agriculture, Guangxi University, Nanning 530004, China; jdhuang@gxu.edu.cn; 4 Hubei Key Laboratory of Food Crop Germplasm and Genetic Improvement, Institute of Food Crops, Hubei Academy of Agricultural Sciences, Wuhan 430064, China; zzlin2022@hbaas.com; 5 School of Life Sciences, Hubei University, Wuhan 430062, China; dtcai8866@163.com

**Keywords:** rice, nitrogen, planting density, grain yield, nitrogen use efficiency

## Abstract

Research indicates that, owing to the enhanced grain-filling rate of tetraploid rice, its yield has notably improved compared to previous levels. Studies conducted on diploid rice have revealed that optimal planting density and fertilization rates play crucial roles in regulating rice yield. In this study, we investigated the effects of different nitrogen application and planting density treatments on the growth, development, yield, and nitrogen utilization in tetraploid (represented by T7, an indica–japonica conventional allotetraploid rice) and diploid rice (Fengliangyou-4, represented by FLY4, a two-line super hybrid rice used as a reference variety for the approval of super rice with a good grain yield performance). The results indicated that the highest grain-filling rate of T7 could reach 77.8% under field experimental conditions due to advancements in tetraploid rice breeding. This is a significant improvement compared with the rate seen in previous research. Under the same conditions, T7 exhibited a significantly lower grain yield than FLY4, which could be attributed to its lower grain-filling rate, spikelets per panicle, panicle number m^−2^, and harvest index score. Nitrogen application and planting density displayed little effect on the grain yield of both genotypes. A higher planting density significantly enhanced the leaf area index and biomass accumulation, but decreased the harvest index score. Compared with T7, FLY4 exhibited a significantly higher nitrogen use efficiency (NUE_g_), which was mainly due to the higher nitrogen content in the straw. Increasing nitrogen application significantly decreased NUE_g_ due to its minimal effect on grain yield combined with its significant enhancement of nitrogen uptake. Our results suggest that the yield and grain-filling rate of T7 have been improved compared with those of previously tested polyploid rice, but are still lower than those of FLY4, and the yield of tetraploid rice can be further improved by enhancing the grain-filling rate, panicle number m^−2^, and spikelets per panicle via genotype improvement.

## 1. Introduction

Rice (*Oryza sativa* L.) is the staple food for over half of the world’s population [1]. It has been estimated that rice production must be increased by 1.2% annually to meet the demand of the rapidly growing population for food in the coming decades [2]. Rice production in China has doubled in the past 50 years, which can be primarily attributed to the development of high-yielding varieties and the improvement in crop management techniques, particularly in terms of nitrogen (N) fertilizer application and irrigation [3,4]. In recent years, intensified climate change and exacerbated global warming, which foster frequent extreme weather events and the emergence of novel pests and diseases, have significantly increased the challenge [5]. Therefore, there is an urgent need to develop new high-yielding crop varieties and further enhance crop productivity to solve the pressing problem of global food shortages [6,7].

Polyploidy is a prevalent phenomenon in plant evolution. It is mainly caused by whole-genome replication or interspecific hybridization. It has been demonstrated that almost all eukaryotes undergo polyploidization during evolution [8]. Compared with diploid plants, polyploid plants have larger leaves and higher levels of nutrients and secondary metabolites [9,10]. Moreover, polyploids often have significant resilience to environmental changes in biomass, vitality, and adaptability [11,12,13], and are therefore widely used in plant breeding. During the evolutionary process from diploids to polyploids, crops such as wheat, cotton, and rapeseed have undergone significant increases in yield. Therefore, researchers have proposed a new strategy for breeding super rice by taking the advantages of both distant hybridization and polyploidy [14]. Homologous tetraploid rice was extensively studied before the 1950s, but the research work ultimately came to a standstill due to difficulties solving the problem of a low grain-filling rate [15,16]. Compared with diploid rice, polyploid rice has various advantages such as a large grain size, a high grain weight, a strong stem, and strong stress resistance. However, improving its yield is very challenging due to its smaller number of panicles per plant, its fewer grains per panicle, and its lower grain-filling rate [17,18,19,20]. Afterwards, breeding with lines enabling polyploid meiosis stability (*PMeS*) gene in rice significantly enhanced the grain-filling rate of polyploid rice. The *PMeS* lines have stable meiosis and a grain-filling rate over 70%, while the HN2026-4X lines have a grain-filling rate over 80%, which contributes to a substantial increase in yield [21,22]. These achievements make the effective production and application of polyploid rice possible. Therefore, it is pivotal to comparatively analyze the distinct mechanisms underlying the yield formation and composition of polyploid and diploid rice for the breeding of high-yield polyploid rice.

N fertilizer application and planting density, which play pivotal roles in regulating nitrogen utilization efficiency (NUE) while mitigating environmental losses [23], are crucial management strategies with significant impacts on rice growth and yield formation [24,25,26]. N is one of the most pivotal nutrients for crop growth and yield, and is the primary nutrient that constrains rice yield [27]. N application can enhance the development and proliferation of the meristematic tissues of plants at the vegetative growth phase, foster the emergence and growth of tillers, and ultimately increase the number of panicles at the mature stage [25,28,29]. At the reproductive growth stage, N fertilization can facilitate panicle development and increase the number of spikelets per panicle, thereby contributing to yield potential [30,31]. However, excessive N fertilization will not only reduce the NUE, but also cause environmental pollution. Planting density has great impacts on rice population structure and biomass accumulation, and therefore is a determinant factor that influences the yield potential of rice [32]. An optimal planting density can improve the population structure, facilitate the efficient utilization of solar radiation, and modulate tillering and grain yield formation in rice [25,33,34]. However, previous research on tetraploid rice has predominantly focused on genetic breeding, and there has been a notable dearth of studies examining the cultivation of tetraploid rice under field conditions. The exploration of optimal N fertilizer management and planting density for polyploid rice can effectively enhance the tillering and panicle number m^−2^, thereby maximizing its yield potential.

In this study, we selected allopolyploid and diploid two-line hybrid rice with good grain-filling rates and yield performances to (1) compare the growth, development, and yield composition of diploid and tetraploid rice; (2) investigate the impacts of N fertilizer management and planting density on the growth and development of both diploid and tetraploid rice; and (3) clarify the influence of N fertilizer management and planting density on the NUE of both diploid and tetraploid rice.

## 2. Materials and Methods

### 2.1. Site Description

Field experiments were conducted at Xinhu Village (30°47′ N, 114°25′ E), Wuhan City, Hubei Province, China during the rice-growing season from May to October in 2018 and 2019. Before fertilizer application, soil samples were collected from the surface layer (0–20 cm layer) of the experimental field for analysis. The soil properties over the past two years were a pH of 5.9, an organic matter of 31.1 g kg^−1^, a total N of 1.9 g kg^−1^, an Olsen-P of 16.1 mg kg^−1^, and an available K of 204.0 mg kg^−1^. The climate data were collected from the meteorological station (AWS 800, Campbell Scientific, Inc., Logan, UT, USA) of Huazhong Agricultural University. During the rice-growing season, the average daily minimum temperature, maximum temperature, and solar radiation were 22.3 °C, 30.9 °C, and 17.4 MJ m^−2^ d^−1^ in 2018, and 22.5 °C, 31.4 °C, and 17.1 MJ m^−2^ d^−1^ in 2019, respectively.

### 2.2. Experimental Design and Crop Management

Two years of experiments were conducted in a split–split plot design with different N application rates as the main plots, planting densities as the subplots, and varieties as the sub-subplots. The experiment included three replications, and the plot area was 24 m^−2^ (4 m × 6 m). The indica–japonica conventional allotetraploid rice T7 was provided by the Wuhan Polyploid Rice Company, and indica two-line super hybrid diploid rice Fengliangyou-4 (FLY4, used as a reference variety for the approval of super rice with a good grain yield performance) was selected for experiments.

The three N (urea) application rates were 150, 225, and 300 kg N ha^−1^, with N1, N2, and N3 indicating low, medium, and high N application rates, respectively. N was applied with 50% at the basal stage, 25% at the mid-tillering stage, and 25% at the panicle initiation (PI) stage. The same amounts of phosphorus (calcium superphosphate) and potassium (potassium chloride) were applied in each plot. Phosphorus, at 75 kg P_2_O_5_ ha^−1,^ was applied at the basal stage, and potassium at 180 kg K_2_O ha^−1^ was applied with a split of 50% at the basal stage and 50% at the PI stage. Fertilizer treatment was consistently maintained for two years. Two transplanting densities were set, namely, a high-planting-density treatment (TD25) with a row spacing of 13.3 cm × 30.0 cm and the planting of 4–6 seedlings per hill, with 25 hills per m^−2^, and a lower-planting-density treatment (TD17) with a row spacing of 20 cm × 30.0 cm and the planting of 6–7 seedlings per hill, with 16.7 hills per m^−2.^ This was performed for two years.

Pre-germinated seeds were sown in seedbeds on 1 June and 26 May in 2018 and 2019, respectively. Transplanting was carried out on 2 July and 23 June in 2018 and 2019, respectively. The soil was plowed and puddled before transplantation. Fertilizer was applied to the plot one day before the transplanting. In order to reduce leakage between plots, all bunds were covered with plastic film, inserted into the soil surface at a depth of more than 20 cm. The field was kept flooded from transplantation until 10 days before maturity, except for the draining and sun drying of the field for one week before the maximum tillering period. Pests, diseases, and weeds were intensively controlled to avoid yield loss.

### 2.3. Sampling and Measurement

#### 2.3.1. Sampling during Rice Growth and Development

Twelve hills of rice plants were sampled at the PI stage, heading (HD) stage, and (MA) mature stage, respectively. The plants were separated into straws, leaves, and panicles when present. The stem number was counted, the area of green leaf blades was measured with a Li-Cor area meter (LI-3100 C, LI-COR, Lincoln, NE, USA), and the leaf area index (LAI) value was calculated. The dry matter of each part was determined after oven drying at 80 °C to a constant weight. Some indicators of dry matter accumulation and transport were calculated according to the following formulas:

Dry matter accumulation post-anthesis (DAP) = total aboveground dry matter accumulation at MA stage − total aboveground dry matter accumulation at HD stage.

Dry matter transport of pre-anthesis (DTP) = grain yield − dry matter accumulation of post-anthesis.

Dry matter transport efficiency of pre-anthesis (DTEP) = dry matter transport of pre-anthesis/total aboveground dry matter accumulation at HD stage.

Dry matter transport contribution rate in pre-anthesis to yield (DTCP) = dry matter transport of pre-anthesis/grain yield.

#### 2.3.2. Dynamic Changes in Tillers

After being transplanted in the field, 12 hills were selected at designated locations in each plot, and the number of tillers was recorded approximately every 7 days until the rice panicles were fully tillered.

#### 2.3.3. SPAD Value Measurement

A chlorophyll meter (SPAD-502 PLUS) produced by the Konica Minolta Corporation in Tokyo, Japan was used to measure the leaf SPAD at the HD stage and 21 days after HD. Six representative flag leaves were selected from each plot and measured at 1/2 of the leaf length, 1/4 of the leaf width, and 3 cm above and below. Each leaf was measured at three points, and the average of three SPAD values was calculated as the SPAD value for this leaf.

#### 2.3.4. Gas Exchange Measurement

Under the conditions of a leaf chamber temperature of 30 °C, a carbon dioxide concentration of 400 µ mol mol^−1^, and a photosynthetic photon flux density (PPFD) of 1500 µ mol m^−2^ s^−1^, the gas exchange of plant leaves was measured in the field using a portable gas exchange system (Li-6800, LI-COR, Lincoln, NE, USA) equipped with a 6 cm^2^ chamber to calculate the net photosynthetic rate of leaves in a sunny and cloudless morning. The inverted 1.5th leaf (the youngest fully expanded leaves) was selected in each plot during the PI stage. The flag leaf was selected in each plot during the booting (BT) stage and HD stage. Each plot was tested four times repeatedly.

#### 2.3.5. Measurement of Grain Yield and Yield-Related Traits

At maturity, the grain yield was determined from a 5 m^−2^ area in each plot and adjusted to a moisture content of 0.14 g H_2_O g^−1^ in terms of fresh weight. Grain moisture content was measured with a digital moisture tester (DMC-700, Seedburo, Chicago, IL, USA). Twelve hills were sampled from each plot to measure the aboveground total biomass, harvest index score, and yield components. Plant samples were separated into straws and panicles after recording the panicle number. The dry weight of straw was determined after oven drying at 80 °C to a constant weight. Panicles were hand-threshed and the fully filled spikelets were separated from unfilled spikelets by being submerged in tap water. Unfilled spikelets were separated from partially filled spikelets via wind selection. Three subsamples containing 30 g of fully filled spikelets, three subsamples containing 2 g of unfilled spikelets, and the entire partially filled sample were taken to count the number of spikelets. The dry weights of rachis, fully filled, partially filled, and unfilled spikelets were determined after oven drying at 80 °C to a constant weight. The aboveground total biomass, panicle number per m^2^, spikelets per panicle, grain-filling percentage, and harvest index value were calculated.

#### 2.3.6. Nitrogen Uptake

We determined the plant N concentration of the samples for yield components using an elemental analyzer (Elementar Vario MAX CNS/CN, Elementar Trading Co., Ltd., Langenselbold, Germany). The N content of each component was the product of N concentration and dry weight. The aboveground total N uptake at the maturity was the sum of the N contents in all components. The N utilization efficiency for grain production (NUE_g_) was calculated according to the following formula:

NUE_g_ = grain yield/total N uptake at maturity

### 2.4. Statistical Analysis

Statistical data analysis was performed using analysis of variance (Statistix 9.0, Analytical Software, Tallahassee, FL, USA). Mean values were compared by the least significance difference (LSD) test at a 0.05 probability level.

## 3. Results

### 3.1. Grain Yield and Yield Components

**Figure 1:**
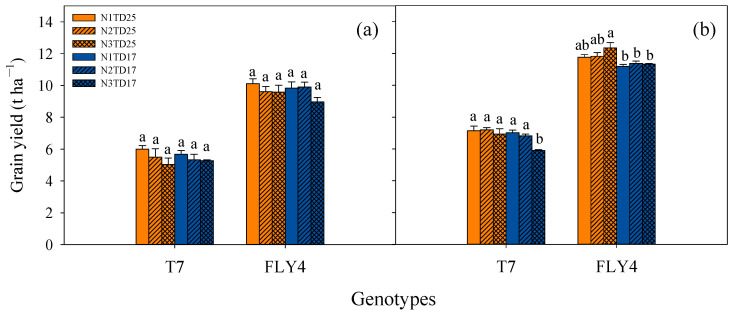
Effects of different planting density and nitrogen application treatments on grain yield in tetraploid T7 and diploid FLY4 rice in 2018 (**a**) and 2019 (**b**). The error bar indicates SE (*n* = 3). Within a group of the same genotypes, different letters indicate significant differences according to LSD (0.05). T7: tetraploid rice; FLY4: Fengliangyou-4. TD17: lower-density treatment (20.0 cm × 30.0 cm), 16.7 hills per m^−2^; TD25: high-density treatment (13.3 cm × 30.0 cm), 25 hills per m^−2^. N1: N rate 150 kg ha^−1^; N2: N rate 225 kg ha^−1^; N3: N rate 300 kg ha^−1^. N1TD25, N1TD17, N2TD25, N2TD17, N3TD25, and N3TD17 represent different combinations of N application rates and density treatments.

The average gain yield of T7 (6.16 t ha^−1^) was significantly lower than that of FLY4 (10.66 t ha^−1^) across the two years (Figure 1). Both genotypes had higher grain yields in 2019 than in 2018. Notably, no significant difference was observed in the grain yield of both genotypes among different N application rates and planting densities in 2018. In 2019, N2TD25 resulted in the highest grain yield of T7 (7.20 t ha^−1^), while N3TD17 led to a significantly lower grain yield (5.90 t ha^−1^) of T7 than other treatments. Under TD25, there was no significant difference in grain yield among different N application rates; while under TD17, N3 treatment significantly reduced the grain yield. In 2019, the highest grain yield of FLY4 was achieved under N3TD25 (12.36 t ha^−1^) treatment. Under the same planting density at different N application rates, there was no significant difference in the grain yield of FLY4; however, TD25 treatment resulted in a significantly higher grain yield than TD17 treatment (Figure 1).

T7 had significantly lower values of all yield components than FLY4, except for grain weight (Table 1). Notably, the highest grain-filling rates of T7 were 73.7% and 77.8% in 2018 and 2019, respectively. Although the average grain-filling rate of T7 (73.9%) across the two years was lower than that of FLY4 (88.7%), it had already reached a high level for tetraploid rice. Moreover, there were no significant differences in yield components under different N application rates and planting densities for both genotypes in 2018, except for panicle number m^−2^, which was higher under TD25 than under T17 treatment. In 2019, TD25 treatment resulted in a significantly larger panicle number m^−2^ and significantly fewer spikelets per panicle than TD17 in both genotypes. In addition, T7 showed decreases in spikelets per panicle with the increasing N application rate, and TD25 resulted in a lower grain weight than TD17 (Table 1).

In 2019, N3TD17 resulted in a significantly lower grain yield of T7 than N1TD17 and N2TD17, which was mainly attributed to having fewer spikelets per panicle; TD25 resulted in a higher grain yield of T7 than TD17, which was mainly due to a higher panicle number m^−2^ and grain weight. In addition, TD25 resulted in a higher grain yield of FLY4 than TD17, which could be mainly attributed to the higher panicle number m^−2^ (Figure 1, Table 1).

### 3.2. Nitrogen Uptake and NUE_g_

There was no significant difference in total N uptake at the MA stage between T7 (187.0 kg ha^−1^) and FLY4 (195.2 kg ha^−1^) in 2018, but FLY4 (182.2 kg ha^−1^) had a significantly higher total N uptake than T7 (163.7 kg ha^−1^) in 2019 (Table 2). At the MA stage, T7 had a significantly higher straw N content and uptake but a significantly lower grain N uptake and NUE_g_ than FLY4 across the two years. Moreover, T7 showed a significantly higher grain N content than FLY4 in 2018, but there was no significant difference between the two genotypes in 2019.

The NUE_g_ of T7 and FLY4 both significantly decreased with an increasing N application rate, and there was no significant difference under different planting densities across the two years (Table 2). For T7, an increasing N application rate led to increases in the total N uptake, straw N uptake, grain N content, and straw N content, while showed little effect on the grain N uptake. Compared with TD17, TD25 resulted in significantly higher total N uptake and straw N uptake for T7, while there was no significant difference in grain N content and straw N content, and the performance was consistent over the two years. There was no significant difference in the grain N uptake of T7 between two planting density treatments in 2018, and TD25 resulted in a higher grain N uptake than TD17 in 2019.

For FLY4, the total N uptake, straw N uptake, straw N content, grain N content, and grain N uptake increased significantly with the increasing N application rate in both years, except for grain N content and uptake in 2018 and straw N content and uptake in 2019, whose differences did not reach the significance level. TD25 resulted in higher straw N uptake than TD17 in 2018, but there was no significant difference under two planting densities in 2019. Compared with TD17, TD25 led to significantly higher total N uptake and grain N uptake, but no significant differences in grain N content and straw N content, and the performance was consistent over the two years (Table 2).

### 3.3. Dynamics of Tillering, Growth, and Development of Tetraploid Rice

**Figure 2:**
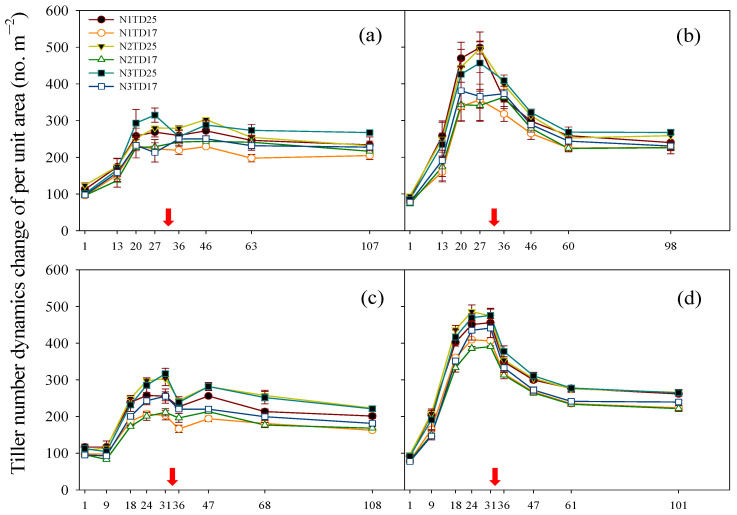
Changes in tillering dynamics in tetraploid T7 (**a**,**c**) and diploid FLY4 (**b**,**d**) under different densities and nitrogen application treatments in 2018 (**a**,**b**) and 2019 (**c**,**d**). The error bar indicates SE (*n* = 3). The red arrow indicates the time point of the panicle initiation stage. TD17: lower-density treatment (20.0 cm × 30.0 cm), 16.7 hills per m^−2^; TD25: high-density treatment (13.3 cm × 30.0 cm), 25 hills per m^−2^. N1: N rate 150 kg ha^−1^; N2: N rate 225 kg ha^−1^; N3: N rate 300 kg ha^−1^. N1TD25, N1TD17, N2TD25, N2TD17, N3TD25 and N3TD17 represent different combinations of N application rates and density treatments.

There were significant differences in the dynamics of tillering between T7 and FLY4 (Figure 2). After rice transplantation, the tiller number m^−2^ of FLY4 first showed an increasing trend, then a decreasing trend, and finally a stabilizing trend with rice growth in both years. Compared with FLY4, T7 had a slower growth rate of tillers after transplantation, a smaller maximum tiller number m^−2^, and significantly lower panicle number m^−2^ at the MA stage. Moreover, T7 had a significantly higher proportion of mature small panicles than FLY4 over the two years (Table S1). Notably, when T7 entered the PI stage, after the rehydration and application of panicle fertilizer, the tiller number m^−2^ first showed an increase followed by decreases, which was different from the direct decrease observed in FLY4 (Figure 2). The variation pattern was consistent across the two years. Between two planting density treatments, TD25 resulted in a higher tiller number m^−2^ than TD17, while N application treatment exhibited no significant effect on the tiller number m^−2^ of both genotypes over two years (Figure 2).

**Figure 3:**
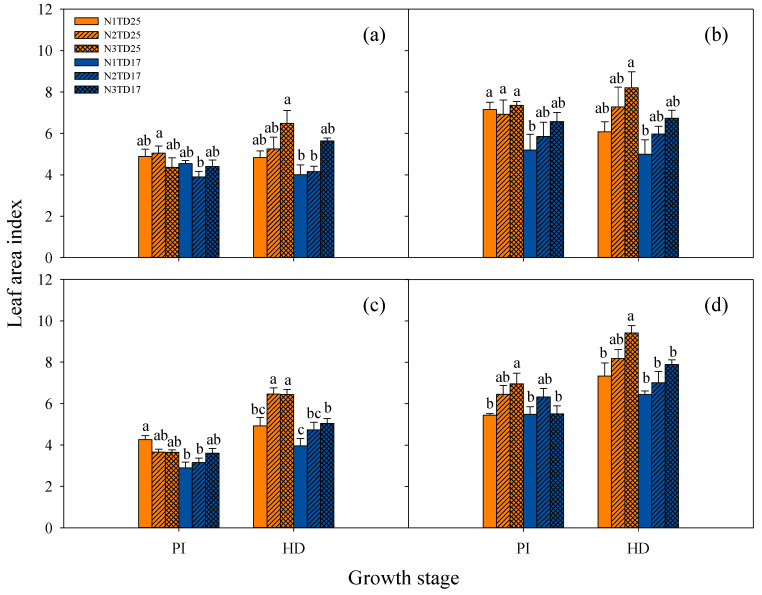
Effects of different planting density and nitrogen application treatments on leaf area index in tetraploid T7 (**a**,**c**) and diploid FLY4 (**b**,**d**) in 2018 (**a**,**b**) and 2019 (**c**,**d**). Error bar indicates SE (*n* = 3). Within a group of the same genotypes, different letters indicate significant differences according to LSD (0.05). TD17: lower-density treatment (20.0 cm × 30.0 cm), 16.7 hills per m^−2^; TD25: high-density treatment (13.3 cm × 30.0 cm), 25 hills per m^−2^. N1: N rate 150 kg ha^−1^; N2: N rate 225 kg ha^−1^; N3: N rate 300 kg ha^−1^. N1TD25, N1TD17, N2TD25, N2TD17, N3TD25 and N3TD17 represent different combinations of N application rates and density treatments. PI: panicle initiation stage approximately 32 days after transplanting; HD: heading stage approximately 63 days after transplanting.

The average LAI across two years of FLY4 (6.27, 7.13) was significantly higher than that of T7 (4.03, 5.16) at both PI and HD stages (Figure 3). At different growth stages, TD25 resulted in a higher LAI than TD17 for both genotypes. There was no consistent pattern in LAI at the PI stage under different N application treatments. However, the LAI at the HD stage showed an increasing trend with an increasing N application rate under the same planting density in both genotypes over the two years.

**Figure 4:**
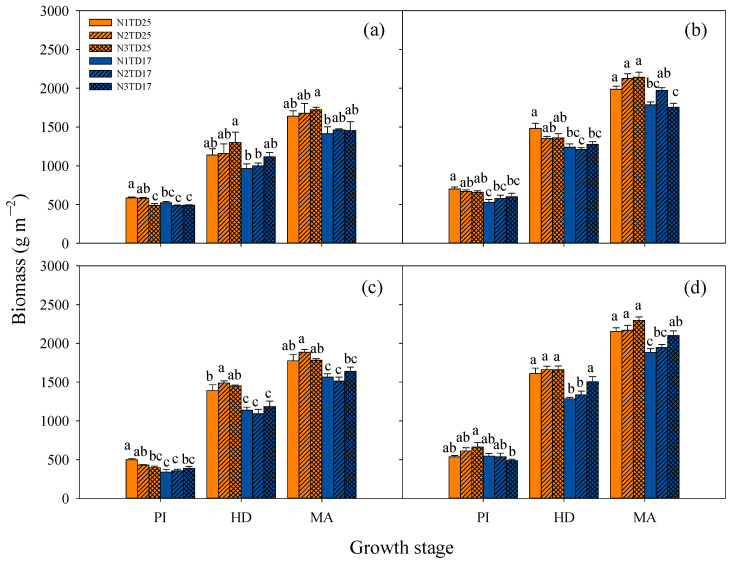
Effects of different planting density and nitrogen application treatments on biomass in tetraploid T7 (**a**,**c**) and diploid FLY4 (**b**,**d**) in 2018 (**a**,**b**) and 2019 (**c**,**d**). The error bar indicates SE (*n* = 3). Within a group of the same genotypes, different letters indicate significant differences according to LSD (0.05). TD17: lower-density treatments (20.0 cm × 30.0 cm), 16.7 hills per m^−2^; TD25: high-density treatments (13.3 cm × 30.0 cm), 25 hills per m^−2^. N1: N rate 150 kg ha^−1^; N2: N rate 225 kg ha^−1^; N3: N rate 300 kg ha^−1^. N1TD25, N1TD17, N2TD25, N2TD17, N3TD25 and N3TD17 represent different combinations of N application rates and density treatments. PI: panicle initiation stage approximately 32 days after transplanting; HD: heading stage approximately 63 days after transplanting; MA: approximately 104 days after transplanting.

In addition, at different growth stages over the two years, TD25 led to higher aboveground dry matter than TD17, while no consistent changing pattern was observed for N application treatments. The average aboveground dry matter across two years of FLY4 (593.2, 1415.0, 2026.7 g m^−2^) at the PI, HD, and MA stages was significantly higher than that of T7 (463.8, 1202.0, 1627.9 g m^−2^) (Figure 4). There were obvious interannual differences in aboveground dry matter at different growth stages. The aboveground dry matter in 2018 was higher at the PI stage but lower at the HD and MA stages compared with that in 2019.

The DAP, DTP, and DTEP of FLY4 were significantly higher than those of T7 in both years (Table 3). The DTCP of T7 was significantly lower than that of FLY4 in 2018, while there was no significant difference in 2019.

### 3.4. Net Photosynthetic Rate and SPAD Value

T7 showed no significant differences in leaf net photosynthetic rate between different N application and planting density treatments across the two years. The net photosynthetic rate of FLY4 was not significantly affected by the N application rate in 2018, but significantly higher under N2 and N3 treatments than under N1 treatment in 2019 (Table 4). At different growth stages, T7 and FLY4 had the highest net photosynthetic rate at the BT stage. The leaf net photosynthetic rate of T7 and FLY4 at the BT and HD stages was lower in 2019 compared with that in 2018. The net photosynthetic rate of T7 leaves was significantly lower than that of FLY4 at the BT stage, but there was no significant difference between the two genotypes at the HD stage. T7 (by 14.6%) showed a less significant decrease in the average net photosynthetic rate from the BT to HD stage than FLY4 (by 20.7%) across the two years.

**Figure 5:**
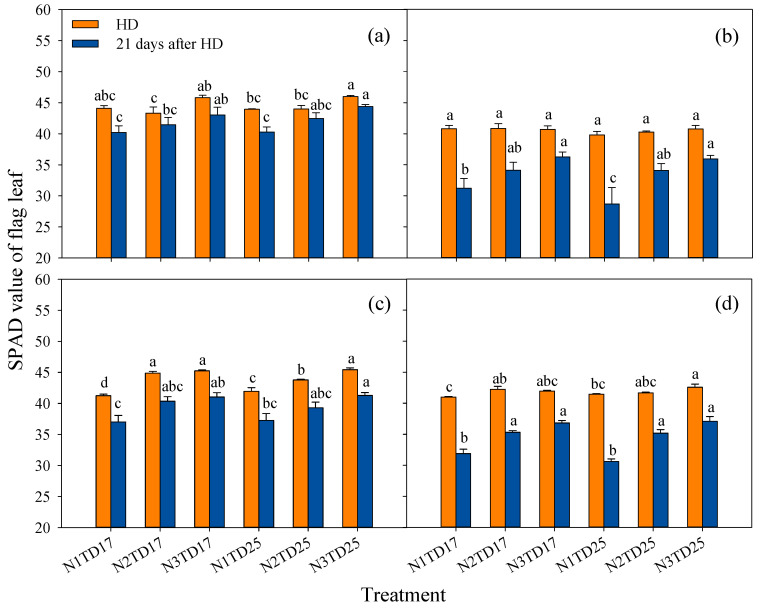
Effects of different treatments on the SPAD value of the flag leaf in tetraploid T7 (**a**,**c**) and diploid FLY4 (**b**,**d**) at HD and 21 days after HD stage in 2018 (**a**,**b**) and 2019 (**c**,**d**). The error bar indicates SE (*n* = 3). In a set of bar charts of the same color, different letters indicate significant differences according to LSD (0.05). Lower-case letters indicate comparisons among different treatments within each group. The content in the legend represents measurement time at HD (approximately 63 days after transplanting) and 21 days after the HD stage. TD17: lower-density treatment (20.0 cm × 30.0 cm), 16.7 hills per m^−2^; TD25: high-density treatment (13.3 cm × 30.0 cm), 25 hills per m^−2^. N1: N rate 150 kg ha^−1^; N2: N rate 225 kg ha^−1^; N3: N rate 300 kg ha^−1^. N1TD25, N1TD17, N2TD25, N2TD17, N3TD25 and N3TD17 represent different combinations of N application rates and density treatments.

Except for the fact that there was no difference in SPAD values between different treatments at the HD stage of FLY4 in 2018, the SPAD values of the flag leaf under all other treatments showed an increasing trend with an increasing N application rate at the HD stage and 21 days after HD (Figure 5). Except for 21 days after HD under N1 in 2018, when the SPAD of FLY4 under TD25 was significantly lower than that under TD17, there were no significant differences among other planting density treatments. At the HD stage and 21 days after HD, the SPAD value of T7 flag leaf was significantly higher than that of FLY4. The two genotypes showed a consistent performance over the two years. The decrease in the SPAD value of both genotypes from the HD stage to 21 days after HD became less significant with an increasing N application rate, and there was no significant difference between different planting density treatments. In addition, T7 (by 7.9%) showed a smaller decrease in the average SPAD value from the HD stage to 21 days after HD across the two years than FLY4 (by 17.6%), and also a slower senescence rate regarding the flag leaf. The change pattern was consistent between the two years.

## 4. Discussion

### 4.1. Comparison of Yield and Yield Components between Diploid and Tetraploid Rice

Polyploidization is one of the primary evolutionary processes driving plant diversification and adaptation [35]. Many plants may have undergone whole-genome duplication events in their evolutionary history [36]. Polyploidization increases genome size and genetic diversity, allowing more favorable genetic variations and stronger adaptability than wild diploid relatives, and has already been successfully implemented in plant breeding programs to increase the overall yield and biomass of several crop species [37]. However, previous research has consistently demonstrated that tetraploid rice exhibits reduced tillering, a smaller panicle number m^−2^, and a lower grain-filling rate, ultimately resulting in a lower overall yield [20,38]. In this study, the average yield of tetraploid rice T7 in two years was 6.16 t ha^−1^, with the highest yield of 7.20 t ha^−1^ under N2TD25 treatment. This was significantly lower than that of diploid rice FLY4, which has an average yield of 10.66 t ha^−1^ in two years (Figure 1). Despite some notable advancements through breeding efforts over an extended period, the problem of a low grain-filling rate has remained unresolved in tetraploid rice [17,39,40]. Also, the average grain-filling rate over a two-year period was a commendable 73.9% and peaked at 77.8% during field experiments in this study. However, the average grain-filling rate of FLY4 reached 88.7% over the two years, with the highest rate achieving 94.0% (Table 1). The low grain-filling rate has been the primary obstacle hindering the utilization of tetraploid rice in the past [41]. Nevertheless, advancement in the discovery and utilization of polyploid meiosis stability (*PMeS*) lines has facilitated the development of numerous polyploid indica–japonica subspecies hybrids, which will help to address the issue of low grain-filling rates in tetraploid rice in the future [21,22,42,43].

The grain-filling process is potentially influenced by the photosynthetic rate of the flag leaf and the efficiency of carbohydrate translocation from the leaf and stem sheath to the developing grains. Here, we observed an obviously higher SPAD value of T7 than that of FLY4 at the HD stage and 21 days after HD, suggesting that T7 may have slower leaf senescence than FLY4 (Figure 5). Photosynthesis is the cornerstone of crop biomass accumulation and yield formation. In this study, we quantitatively assessed the photosynthetic rate of the newest fully expanded leaf at the PI stage, as well as the flag leaf at the BT and HD stages under saturated light conditions (Table 4). These measurements were aimed to provide insights into the photosynthetic performance at different growth stages and its influence on plant growth. T7 showed no significant difference in photosynthetic rate at the HD stage, but a notably lower photosynthetic rate at the PI and BT stages across two years compared with FLY4 (Table 4). Further research should focus on comparing the efficiency of carbohydrate transport from the leaf and stem sheath to the grain between diploid and tetraploid rice after the HD stage. Moreover, leaf photosynthesis frequently undergoes dynamic variations in its state, primarily due to stimulation by external environmental factors. Therefore, more attention should be paid to the influence of this dynamic photosynthesis on biomass accumulation and the grain-filling process in the future. Besides the grain-filling rate, grain yield is also affected by other components, including biomass, the panicle number, the spikelets per panicle, the grain weight, and harvest index values. Although the tetraploid rice T7 exhibited a significantly higher grain weight than diploid rice FLY4 in two years, it exhibited a significantly lower biomass, panicle number m^−2^, and harvest index value, as well as a significantly higher proportion of small panicles than FLY4, which ultimately decreased the grain yield of tetraploid rice (Figure 4; Table 2 and Table S1). Moreover, there was a significant positive correlation between photosynthesis and biomass accumulation (Figure S1).

### 4.2. Effect of Nitrogen Application and Planting Density on the Yield of Diploid and Tetraploid Rice

N application is one the main factors contributing to the increase in rice yield over the past half century [44,45]. Previous research on the cultivation of diploid rice has consistently demonstrated that, with increasing N application, the grain yield initially exhibits an increasing trend and peaks at an application rate of 300 kg N ha^−1^, followed by gradual decreases [46]. In this study, we designed a gradient of N fertilizer treatments, including N1, N2, and N3. However, upon increasing N application, we did not discern the gradual augmentation of grain yield, as the results showed little difference in grain yield among N application treatments in most cases based on the two-year experiments (Figure 1). We even observed a negative trend in yield for tetraploid rice with increasing N application, as the grain yield of T7 under N3 treatment was significantly lower than that under N2 and N1 treatments at a low planting density in 2019 (Figure 1). One possible reason for this observation may be that tetraploid rice has a relatively lower yield level, and thus does not require substantial N fertilizer; hence, an increase in N application may not exert a beneficial effect on its grain yield. Another plausible reason may be the inherent high N content in the soil. N is a pivotal constituent of proteins, chlorophyll, and amino acids, which are intimately associated with the process of photosynthesis. Here, we observed a pronounced increase in chlorophyll content, as measured by SPAD values, in response to increasing N application (Figure 5). However, only minor variations in photosynthetic rate were observed across different N treatments; this may be due to the complexity of the field environment and deserves further investigation (Table 4). An increase in LAI can enhance carbon assimilation, thereby facilitating biomass accumulation under higher N supplies.

Planting density is a pivotal regulatory factor with significant influence on the population structure and grain yield of rice. Generally, rice yield exhibits a positive correlation with the planting density within a specified range. However, once the planting density exceeds the optimal threshold, the yield will demonstrate a declining trend. We set two planting densities, including TD25 and TD17, in this study. However, minimal differences in grain yield were observed between the two planting densities (Figure 1). Several studies have consistently demonstrated that dense planting enhances the LAI and promotes dry matter accumulation in rice across various growth stages, which is consistent with our results. TD25 resulted in significantly higher LAI and biomass accumulation (Figure 3 and Figure 4), as well as a significantly higher panicle number m^−2^ than TD17 (Table 1). However, TD25 led to a marked decline in harvest index value compared with TD17 based on two years of field experiments, which may account for the small difference observed in grain yield between the two planting densities (Table 1). Moreover, no significant difference in photosynthetic rate was observed between the two planting densities (Table 4). TD25 resulted in a higher biomass accumulation rate, which might be mainly due to the higher tiller number and LAI. Therefore, it will be necessary to develop and implement strategies aimed at improving the harvest index value under high-density planting conditions, which is crucial for ensuring sustainable and efficient crop production.

### 4.3. Response of Nitrogen Uptake and Application to Nitrogen and Planting Density Treatments in Tetraploid and Diploid Rice 

In 2018, no statistically significant difference was observed in total N uptake between T7 and FLY4 (Table 2). However, FLY4 demonstrated a significantly higher total N uptake than T7 in 2019. Although FLY4 only exhibited a 7.61% higher average total N uptake at maturity than T7, it showed 73.0% and 24.6% higher grain yield and biomass at maturity compared with T7 (Figure 1 and Figure 4; Table 2). These results indicate that T7 has comparable or even superior N uptake ability compared with FLY4. However, T7 has a limited grain sink capacity and grain-filling rate, resulting in less allocation of assimilates to grain filling and the accumulation of photosynthetic products and N, predominantly in straw (Table 1 and Table 2). Previous research has highlighted that excessive plant N concentration and accumulation can impede the translocation of N from leaf sheaths and stems to panicles, thereby diminishing the grain N accumulation and ultimately decreasing the NUE [47]. The disparity in N uptake between T7 and FLY4 was notably smaller than their difference in yield and biomass, which can be primarily attributed to the higher N concentration and accumulation in its straw (Table 2). This characteristic narrows the overall gap in total N accumulation between the two genotypes, where FLY4 predominantly accumulates N in grains, whereas T7 exhibits substantial N accumulation in the straw (Table 2). The NUE of rice exhibits a significant negative correlation with the N content in stems and leaves at maturity [47]. Thus, compared with that of FLY4, the higher N content observed in the straw of T7 ultimately led to a lower NUE_g_.

In both years, T7 and FLY4 exhibited the highest total N uptake under N3TD25 treatment (Table 2). Some studies have indicated that an increase in planting density, particularly under high N application rates, will contribute to the higher aboveground N accumulation of plants [48,49,50]. With increases in straw N content, straw N uptake, and grain N content in response to N application, the total N uptake at maturity exhibited a significant increase for both FLY4 and T7, demonstrating a positive correlation with N application. However, despite the increase in N application, the grain yield of FLY4 remained largely unchanged, and T7 even exhibited a declining trend in grain yield, resulting in a notable reduction in NUE_g_ for both genotypes (Figure 1, Table 2). This observation is consistent with the previous findings of [46,47], who reported that N uptake in rice plants rises with increasing N supply, whereas the NUE exhibits a declining trend. No significant difference was noted in the grain N content or straw N content over the two-year period, while the enhancement of biomass and grain yield led to increases in N uptake in both straw and grains, ultimately contributing to a higher total N uptake under TD25 treatment (Figure 1 and Figure 4; Table 2). However, NUE_g_ showed no significant differences between planting density treatments.

In summary, an optimal combination of N application and planting density not only elevates grain yield and NUE but also mitigates N input, thereby achieving more sustainable and efficient crop production. Our results indicate that the optimal balance between grain yield and NUE can be attained under the conditions of a low N application rate of 150 kg ha^−1^ coupled with a high planting density of 25 hills per m^−2^.

## 5. Conclusions

Our results demonstrated that the grain-filling rate, panicle number m^−2^, spikelets per panicle, and harvest index value of tetraploid rice T7 are significantly lower than those of diploid rice FLY4, which ultimately results in a lower grain yield of T7 relative to FLY4. N application and planting density treatments showed minimal influence on the grain yield. T7 demonstrated a notably lower NUE_g_ compared with FLY4, primarily due to its higher straw N content. While an increase in N input markedly enhanced the overall N acquisition, it ultimately led to a decline in NUE_g_. Our results indicate that the optimal balance between grain yield and NUE_g_ for both tetraploid and diploid rice can be attained under field conditions of a low N application rate of 150 kg ha^−1^ coupled with a high planting density of 25 hills per m^−2^.

## Figures and Tables

**Table 1:** Effects of different planting densities and nitrogen application rates on yield composition and harvest index value of tetraploid and diploid rice at maturity stage in 2018 and 2019.

Year	Genotypes	Densities	N Rates	Panicle Number (m^−2^)	Spikelets per Panicle	Grain-Filling Rate (%)	Grain Weight (mg)	Harvest Index (%)
2018	T7	TD17	N1	204.6 b	116.1 a	73.7 a	39.1 a	39.3 a
			N2	216.6 ab	107.6 a	71.1 a	38.9 a	36.1 a
			N3	227.8 ab	107.1 a	72.8 a	38.1 a	35.8 a
		TD25	N1	233.8 ab	112.9 a	70.6 a	39.9 a	36.4 a
			N2	231.3 ab	109.9 a	72.9 a	38.5 a	35.1 a
			N3	254.2 a	100.4 a	69.8 a	39.1 a	30.8 b
		Mean		228.0 B	109.0 B	71.8 B	38.9 A	35.6 B
Analysis of variance		N/TD/N × TD		ns/*/ns	ns/ns/ns	ns/ns/ns	ns/ns/ns	*/*/ns
	FLY4	TD17	N1	226.9 bc	186.4 a	85.9 ab	25.2 a	46.6 ab
			N2	225.9 c	190.0 a	85.9 ab	26.2 a	45.9 ab
			N3	230.6 bc	181.2 a	83.4 b	25.5 a	46.4 ab
		TD25	N1	240.0 abc	190.7 a	88.3 a	24.9 a	47.9 a
			N2	258.8 ab	184.1 a	85.8 ab	25.5 a	44.8 b
			N3	267.5 a	185.3 a	85.3 ab	24.8 a	45.2 b
		Mean		241.6 A	186.3 A	85.8 A	25.4 B	46.1 A
Analysis of variance		N/TD/N × TD		ns/**/ns	ns/ns/ns	ns/ns/ns	ns/ns/ns	ns/ns/ns
2019	T7	TD17	N1	162.5 c	163.0 a	75.2 ab	37.1 b	41.1 a
			N2	168.1 c	153.4 ab	75.5 ab	36.7 b	40.8 a
			N3	181.3 bc	132.7 bc	75.9 ab	37.2 b	35.4 c
		TD25	N1	201.1 ab	133.2 bc	73.9 b	38.1 a	37.9 b
			N2	222.9 a	124.1 c	76.9 ab	37.7 ab	36.4 bc
			N3	220.9 a	119.9 c	77.8 a	37.4 ab	37.7 bc
		Mean		192.8 B	137.7 B	75.9 B	37.4 A	38.2 B
Analysis of variance		N/TD/N × TD		ns/**/ns	*/*/ns	ns/ns/ns	ns/*/ns	**/*/**
	FLY4	TD17	N1	223.6 c	198.9 abc	91.9 a	25.3 a	52.8 a
			N2	221.5 c	208.7 a	91.8 a	25.4 a	53.2 a
			N3	239.6 bc	205.4 ab	89.9 a	25.5 a	51.2 ab
		TD25	N1	261.5 ab	176.9 c	94.0 a	25.4 a	49.9 b
			N2	265.6 a	184.4 bc	91.3 a	25.7 a	51.1 ab
			N3	264.6 a	200.6 abc	90.5 a	25.6 a	51.1 ab
		Mean		246.1 A	195.8 A	91.6 A	25.5 B	51.6 A
Analysis of variance		N/TD/N × TD		ns/**/ns	ns/*/ns	ns/ns/ns	ns/ns/ns	ns/*/ns

Within a column for the same genotypes, different letters indicate significant differences according to LSD (0.05). Lower-case and upper-case letters indicate comparisons among different treatments within each group and between means of the two genotypes, respectively. T7: tetraploid rice; FLY4: Fengliangyou-4. TD17: lower-density treatment (20.0 cm × 30.0 cm), 16.7 hills per m^−2^; TD25: high-density treatment (13.3 cm × 30.0 cm), 25 hills per m^−2^. N1: N rate 150 kg ha^−1^; N2: N rate 225 kg ha^−1^; N3: N rate 300 kg ha^−1^. ns, not significant at a 0.05 probability level; * and **, significant at 0.05 and 0.01 probability levels, respectively.

**Table 2:** Effects of different planting densities and nitrogen application rates on nitrogen uptake and utilization in different parts of tetraploid and diploid rice at maturity in 2018 and 2019.

Year	Genotypes	Densities	N Rates	Straw N Content (%)	Grain N Content (%)	Straw N Uptake (kg ha^−1^)	Grain N Uptake (kg ha^−1^)	Total N Uptake (kg ha^−1^)	NUE_g_ (kg kg^−1^)
2018	T7	TD17	N1	1.06 b	1.28 b	74.7 d	70.5 a	162.9 d	34.2 a
			N2	1.06 b	1.34 ab	82.0 cd	70.8 a	170.0 cd	31.2 ab
			N3	1.35 a	1.47 a	102.0 ab	76.4 a	200.3 ab	26.0 bc
		TD25	N1	1.00 b	1.33 b	85.4 bcd	79.3 a	184.2 bcd	32.4 a
			N2	1.05 b	1.36 ab	95.4 bc	80.7 a	193.3 abc	30.6 abc
			N3	1.18 ab	1.35 ab	116.4 a	71.4 a	211.6 a	25.2 c
		Mean		1.12 A	1.36 A	92.7 A	74.9 B	187.0 A	29.9 B
Analysis of variance		N/TD/N × TD		*/ns/ns	*/ns/ns	*/*/ns	ns/ns/ns	*/*/ns	**/ns/ns
	FLY4	TD17	N1	0.55 bc	1.24 b	46.1 d	103.4 c	160.4 c	52.1 a
			N2	0.75 a	1.36 a	72.7 abc	123.6 ab	206.4 ab	43.9 b
			N3	0.81 a	1.34 ab	66.4 bcd	109.0 bc	189.1 bc	43.1 b
		TD25	N1	0.53 c	1.23 b	49.5 cd	117.5 abc	175.5 c	54.4 a
			N2	0.70 ab	1.31 ab	73.3 ab	125.2 a	211.6 ab	45.0 b
			N3	0.80 a	1.33 ab	83.5 a	129.3 a	228.0 a	42.6 b
		Mean		0.69 B	1.30 B	65.3 B	118.0 A	195.2 A	46.9 A
Analysis of variance		N/TD/N × TD		*/ns/ns	*/ns/ns	*/*/ns	ns/*/ns	**/*/ns	**/ns/ns
2019	T7	TD17	N1	0.63 d	1.12 ab	48.5 c	72.0 b	134.3 c	47.9 a
			N2	0.87 ab	1.19 ab	64.9 b	73.4 b	153.5 b	40.3 bc
			N3	0.95 a	1.22 a	86.1 a	70.6 b	173.2 a	33.6 d
		TD25	N1	0.68 cd	1.10 b	64.1 b	74.0 b	152.2 b	44.3 ab
			N2	0.77 bc	1.21 ab	79.1 a	82.7 a	179.1 a	38.3 cd
			N3	0.97 a	1.20 ab	92.3 a	80.7 a	189.7 a	35.6 cd
		Mean		0.81 A	1.17 A	72.5 A	75.6 B	163.7 B	40.0 B
Analysis of variance		N/TD/N × TD		**/ns/ns	*/ns/ns	**/**/ns	ns/**/*	*/**/ns	**/ns/ns
	FLY4	TD17	N1	0.48 a	1.13 b	38.9 b	112.7 d	157.8 c	63.9 a
			N2	0.48 a	1.20 ab	39.6 b	123.9 bcd	170.1 bc	61.2 ab
			N3	0.53 a	1.27 a	48.9 ab	135.7 b	194.1 a	55.6 b
		TD25	N1	0.43 a	1.14 b	43.0 ab	122.6 cd	171.3 bc	62.7 ab
			N2	0.52 a	1.21 ab	50.1 a	133.8 bc	191.0 ab	58.2 ab
			N3	0.48 a	1.28 a	48.7 ab	150.3 a	208.6 a	56.3 ab
		Mean		0.49 B	1.21 A	44.9 B	129.8 A	182.2 A	59.6 A
Analysis of variance		N/TD/N × TD		ns/ns/ns	*/ns/ns	*/ns/ns	**/*/ns	**/*/ns	*/ns/ns

Within a column for the same genotypes, different letters indicate significant differences according to LSD (0.05). Lower-case and upper-case letters indicate comparisons among different treatments within each group and between the means of the two genotypes, respectively. T7: tetraploid rice; FLY4: Fengliangyou-4. TD17: lower-density treatment (20.0 cm × 30.0 cm), 16.7 hills per m^−2^; TD25: high-density treatment (13.3 cm × 30.0 cm), 25 hills per m^−2^. N1: N rate 150 kg ha^−1^; N2: N rate 225 kg ha^−1^; N3: N rate 300 kg ha^−1^. ns, not significant at 0.05 probability level; * and **, significant at 0.05 and 0.01 probability levels, respectively. NUE_g_: N utilization efficiency for grain production.

**Table 3:** Differences in dry matter accumulation and transport between tetraploid and diploid rice in 2018 and 2019.

Year	Genotypes	DAP (g m^−2^)	DTP (g m^−2^)	DTEP (%)	DTCP (%)
2018	T7	438.3 b	122.1 b	10.1 b	22.7 b
	FLY4	642.0 a	387.2 a	29.0 a	37.9 a
2019	T7	402.5 b	243.0 b	18.3 b	37.5 a
	FLY4	581.3 a	495.2 a	32.6 a	45.9 a

A small panicle refers to a panicle with less than half the average number of spikelets per panicle when mature. Within a column referring to the same year, different lower-case letters indicate significant differences according to LSD (0.05) among two genotypes. DAP: dry matter accumulation post-anthesis; DTP: dry matter transport pre-anthesis; DTEP: dry matter transport efficiency of pre-anthesis; DTCP: dry matter transport contribution rate to yield in pre-anthesis stage. T7: tetraploid rice; FLY4: Fengliangyou-4.

**Table 4:** Changes in net photosynthetic rates during different growth periods in tetraploid and diploid rice under different treatments in 2018 and 2019.

Year	Genotypes	Densities	N Rates	PI-NPR (µmol m⁻^2^s⁻^1^)	BT-NPR (µmol m⁻^2^s⁻^1^)	HD-NPR (µmol m⁻^2^s⁻^1^)
2018	T7	TD17	N1	26.2 a	27.8 a	24.1 a
			N2	26.1 a	29.1 a	22.3 a
			N3	27.7 a	28.2 a	24.4 a
		TD25	N1	24.3 a	27.4 a	23.6 a
			N2	25.7 a	27.9 a	22.4 a
			N3	26.1 a	29.4 a	23.7 a
		Mean		26.0 B	28.3 B	23.4 A
Analysis of variance		N/TD/N × TD		ns/ns/ns	ns/ns/ns	ns/ns/ns
	FLY4	TD17	N1	31.9 a	31.1 ab	24.8 a
			N2	28.3 a	33.8 a	24.1 a
			N3	28.7 a	29.6 b	24.0 a
		TD25	N1	31.7 a	32.7 ab	21.6 a
			N2	29.0 a	30.3 ab	25.4 a
			N3	28.7 a	29.6 b	22.9 a
		Mean		29.7 A	31.2 A	23.8 A
Analysis of variance		N/TD/N × TD		ns/ns/ns	ns/ns/ns	ns/ns/ns
2019	T7	TD17	N1		23.6 a	21.3 a
			N2		25.0 a	20.9 a
			N3		25.1 a	22.1 a
		TD25	N1		23.8 a	21.1 a
			N2		24.9 a	22.0 a
			N3		24.3 a	22.3 a
		Mean			24.5 B	21.6 A
Analysis of variance		N/TD/N × TD			ns/ns/ns	ns/ns/ns
	FLY4	TD17	N1		24.9 bc	20.1 b
			N2		26.8 ab	23.4 a
			N3		27.3 ab	21.5 ab
		TD25	N1		22.8 c	19.6 b
			N2		26.2 ab	22.4 ab
			N3		28.5 a	22.1 ab
		Mean			26.1 A	21.5 A
Analysis of variance		N/TD/N × TD			*/ns/ns	*/ns/ns

Within a column for the same genotypes, different letters indicate significant differences according to LSD (0.05). Lower-case and upper-case letters indicate comparisons among different treatments within each group and the between means of the two genotypes, respectively. T7: tetraploid rice; FLY4: Fengliangyou-4. TD17: lower-density treatment (20.0 cm × 30.0 cm), 16.7 hills per m^−2^; TD25: high-density treatment (13.3 cm × 30.0 cm), 25 hills per m^−2^. N1: N rate 150 kg ha^−1^; N2: N rate 225 kg ha^−1^; N3: N rate 300 kg ha^−1^. ns, not significant at 0.05 probability level; * significant at 0.05 probability levels. PI-NPR, BT-NPR and HD-NPR represents the net photosynthetic rates during the PI stage, which is approximately 32 days after transplanting; the BT stage, which is approximately 52 days after transplanting, and the HD stage, which is approximately 63 days after transplanting, respectively.

## Data Availability

The datasets collected and/or analyzed in the present study are available from the corresponding author upon reasonable request.

## Supplementary Materials

The following supporting information can be downloaded at: https://www.mdpi.com/article/10.3390/plants13202884/s1, Figure S1: Correlations between net photosynthetic rate and biomass during maturity in 2018 (a) and 2019 (b). The black solid circle represents net photosynthetic rate during the BT stage vs. mature biomass, white hollow circle represents net photosynthetic rate during the HD stage vs. mature biomass. BT: approximately 52 days after transplanting, HD: approximately 63 days after transplanting; Table S1: Proportion of small panicles in tetraploid and diploid rice at maturity under different planting density and nitrogen application treatments in 2018 and 2019.
