# Supplementary material for: Differences in Grain Yield and Nitrogen Uptake between Tetraploid and Diploid Rice: The Physiological Mechanisms under Field Conditions
Source: Plants (Basel). 2024 Oct 15;13(20):2884. doi: 10.3390/plants13202884 (PMC11510817; doi:10.3390/plants13202884)
Supplement: Supplementary file 1 [file plants-13-02884-s001.zip › plants-3238416-supplementary.pdf]

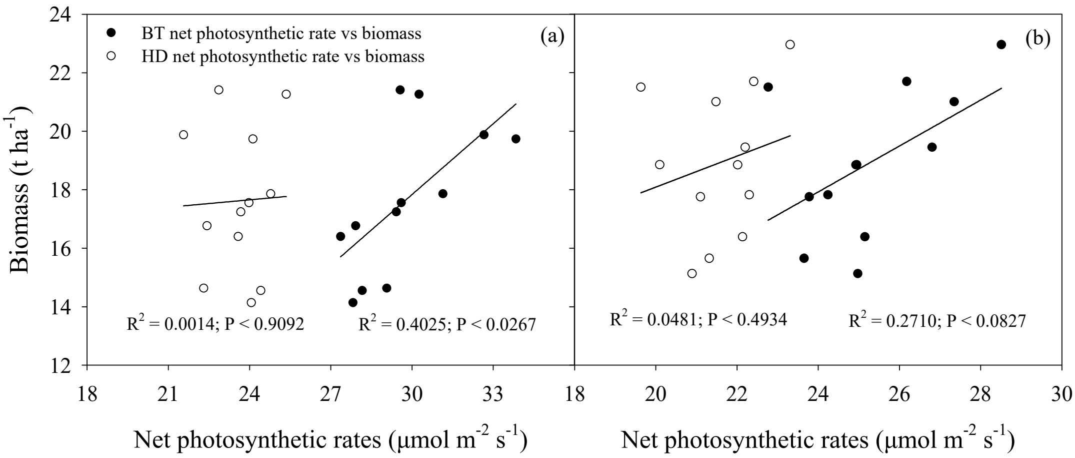

Figure S1. Correlations between net photosynthetic rate and biomass during maturity in 2018 (a) and 2019 (b). The black solid circle represents net photosynthetic rate during the BT stage *vs* mature biomass, white hollow circle represents net photosynthetic rate during the HD stage *vs* mature biomass. BT: approximately 52 days after transplanting, HD: approximately 63 days after transplanting.

Table S1 Proportion of small panicles in tetraploid and diploid rice at maturity under different planting density and nitrogen application treatments in 2018 and 2019.

Genotypes	Densities	N rates	Proportion of small panicle (%)	
			2018	2019
T7	TD17	N1	18.2 a	15.8 a
		N2	24.3 a	21.1 a
		N3	20.2 a	17.6 a
	TD25	N1	20.9 a	18.2 a
		N2	23.7 a	20.9 a
		N3	22.5 a	19.5 a
	Mean		21.6 A	18.8 A
FLY4	TD17	N1	6.7 a	6.4 a
		N2	8.0 a	8.4 a
		N3	8.5 a	8.7 a
	TD25	N1	8.6 a	9.6 a
		N2	7.8 a	8.0 a
		N3	8.2 a	7.4 a
	Mean		8.0 B	8.1 B

Small panicle refers to a panicle with less than half the average number of spikelets per panicle. Within a column for the same genotypes, different letters indicate significant difference according to LSD (0.05). Lower-case and upper-case letters indicate comparisons among different treatments within each group and between means of the two genotypes, respectively. T7: tetraploid rice, FLY4: Fengliangyou-4. TD17: lower density treatment (20.0 cm × 30.0 cm), 16.7 hills per m^{-2}, TD25: high density treatment (13.3 cm × 30.0 cm), 25 hills per m^{-2}. N1: N rate 150 kg ha^{-1}, N2: N rate 225 kg ha^{-1}, N3: N rate 300 kg ha^{-1}.
